# Formative Evaluation of a Comprehensive Self-Management Intervention for Irritable Bowel Syndrome, Comorbid Anxiety, and Depression: Mixed Methods Study

**DOI:** 10.2196/43286

**Published:** 2024-01-31

**Authors:** Kendra Kamp, Pei-Lin Yang, Emily Friedman, Alejandra Lopez, Sarah Iribarren, Pamela Barney, Sean Munson, Margaret Heitkemper, Rona Levy

**Affiliations:** 1 Department of Biobehavioral Nursing and Health Informatics, School of Nursing University of Washington Seattle, WA United States; 2 National Defense Medical Center Taipei Taiwan; 3 Alacrity Center University of Washington Seattle, WA United States; 4 Human Centered Design and Engineering University of Washington Seattle, WA United States; 5 School of Social Work, University of Washington Seattle, WA United States

**Keywords:** acceptability, anxiety, depression, design, effectiveness, feasibility, implementation, intervention, irritable bowel syndrome, management, mixed methods, patient, self-management, support, usability

## Abstract

**Background:**

Irritable bowel syndrome (IBS) is a disorder of the gut-brain interaction that is associated with abdominal pain, altered bowel patterns, and reduced quality of life. Up to 50% of patients with IBS also report anxiety or depressive symptoms. Although effective self-management interventions exist for individuals with IBS, few have been effectively implemented, and most do not consider the unique needs of patients with comorbid IBS and anxiety or depression.

**Objective:**

This study aimed to determine the anticipated acceptability, appropriateness, feasibility, and usability of a comprehensive self-management intervention using an implementation science and human-centered design approach among individuals with comorbid IBS and anxiety or depression and health care providers.

**Methods:**

A convergent mixed methods design was used to elicit feedback on the comprehensive self-management intervention outline and content to identify refinement needs before testing. Patients with IBS and moderate to severe anxiety or depression and health care providers were purposefully sampled from primary care and gastroenterology settings. Participants completed semistructured interviews and surveys on anticipated acceptability, appropriateness, feasibility, and usability.

**Results:**

Patient participants (n=12) were on average 36.8 (SD 12.2) years of age, and 42% (5/12) were currently receiving psychological therapy. Health care providers (n=14) were from primary care (n=7) and gastroenterology (n=7) settings. The mean usability scores (out of 100) were 52.5 (SD 14.5) for patients and 45.6 (SD 11.6) for providers. For patients and providers, qualitative data expanded the quantitative findings for acceptability and appropriateness. Acceptability findings were the comprehensive nature of the intervention and discussion of the gut-brain interaction. For appropriateness, participants reported that the intervention provided structure, accountability, and support. Feasibility was confirmed for patients, but there was a divergence of findings between quantitative and qualitative measures for providers. Patients focused on intervention feasibility, while providers focused on implementation feasibility in the clinic. Identified usability issues to address before implementation included the intervention delivery format, length, and lack of integration into health care settings that, if not addressed, may limit the reach of the intervention.

**Conclusions:**

Patients and health care providers found the intervention acceptable and appropriate. Several feasibility and usability issues were identified, including intervention delivery methods, length of intervention, and the best methods to implement in the clinic setting. The next steps are to refine the intervention to address the identified issues and test in a pilot study whether addressing usability issues leads to the anticipated improvements in implementation and uptake.

## Introduction

Irritable bowel syndrome (IBS) is a disorder of gut-brain interaction that affects 6%-18% of individuals worldwide [1,2]. Many evidence-based practice interventions (EBPIs) have been developed to address symptoms of IBS, such as abdominal pain, and improve quality of life [3]. However, a gap exists in translating EBPIs into clinical practice settings. Clinical practice guidelines support the use of behavioral EBPIs for IBS [4,5], yet only a small proportion of patients actually receive such interventions [6]. Implementation science is a field that focuses on translating evidence-based practice into real-world settings [7]. Attending to implementation outcomes such as acceptability, feasibility, appropriateness, and usability can assist in identifying facilitators and barriers to successful intervention implementation and adoption within clinical practice settings [7-9].

When integrating EBPIs for IBS into real-world settings, an important consideration is the common comorbidities that exist among many individuals with IBS. Most notably, 30%-50% of patients diagnosed with IBS also have a diagnosis of anxiety or depression [10]. Psychological distress is linked to the onset and exacerbation of IBS symptoms, and reducing symptom severity and burden is a key component of behavioral approaches. With the publication of the consensus-driven ROME IV criteria [11], there is a growing appreciation that IBS is a disorder of gut-brain interaction. Although many studies have examined the effectiveness of interventions for IBS, including cognitive behavioral therapy and dietary interventions [6,12,13], few have focused specifically on interventions for individuals with IBS and comorbid anxiety or depression. Given the high prevalence of IBS in the United States, along with comorbid anxiety and depression, there is a significant need to implement effective therapeutic strategies to address both IBS and psychological distress [12,14].

One EBPI, the comprehensive self-management (CSM) intervention, has been shown in multiple randomized controlled trials to reduce abdominal pain symptoms and improve quality of life [15-17]. The intervention content has been published as a book, *Master Your IBS* [15-17]. Although initially developed for IBS symptoms, the intervention has elements of a transdiagnostic approach, reducing other common symptoms of anxiety, depression, extraintestinal pain, fatigue, and sleep disturbances [18,19]. The intervention consists of 8 1-hour sessions, which can be provided by a psychiatric nurse practitioner or similarly trained health professional. However, there is a gap in knowledge regarding how to implement the CSM intervention into clinical practice, specifically from the perspective of key stakeholders: individuals with IBS and comorbid anxiety or depression and health care providers.

Research has argued for applying human-centered design and usability principles to address the lack of intervention implementation by redesigning interventions to improve usability while retaining the effective components [20-22]. Although usability has been most often applied in technology-based applications, usability evaluation principles can also be used to assess other products and services, including interventions and implementation strategies [9,23,24]. Human-centered design approaches focus on developing usable interventions through stakeholder input [21]. By addressing design and content issues, interventions can have increased usability and acceptability to better integrate into clinical settings.

In this research, we sought to examine the usability and acceptability of the current, paper-based CSM intervention from the perspectives of patients with IBS and comorbid anxiety or depression and health care providers in primary care and gastroenterology settings. Our formative evaluation was intended to support (1) refining and adapting the CSM to a digital format and (2) the identification of implementation strategies to facilitate adoption in clinical practice settings. The initial impressions patients and health care providers form regarding the anticipated acceptability, feasibility, appropriateness, and usability of an intervention affect their likelihood of adopting it and help characterize their needs, and these impressions can inform intervention refinement or redesign as well as the selection of intervention strategies to plan for integration into clinical practice. Our research questions were as follows: (1) What are patient and provider perspectives on the acceptability, appropriateness, feasibility, and usability of the current CSM intervention? (2) What recommendations do patients and providers have for improving this intervention?

## Methods

### Design

We used a convergent mixed methods design to collect both quantitative (ie, surveys) and qualitative (ie, semistructured interviews) data [25] from patients with IBS and comorbid anxiety or depression, as well as primary care and gastroenterology health care providers. In this study, participants received the paper-based intervention content to review and critique, not the intervention directly. Each patient and health care provider participated in a semistructured interview to discuss the intervention content and structure and answer survey questions. Our interview and survey questions were guided by the discover, design, build, and test framework, which combines perspectives from implementation science and human-centered design [7,8,20]. To gain a comprehensive understanding of issues and recommended strategies, we designed our qualitative interview questions to align with the concepts addressed in the surveys [25]; these included acceptability, feasibility, appropriateness [26], and usability [9] of the CSM intervention content and structure.

### Sample

Individuals with IBS and self-identified comorbid anxiety or depression symptoms (referred to as patients) were recruited on the internet through 2 methods: the University of Washington Institute of Translational Health Sciences listservs and ResearchMatch, a national health volunteer registry supported by the US National Institutes of Health as part of the Clinical Translational Science Award program.

Patients were eligible if they met the following inclusion criteria: (1) aged between 18 and 70 years; (2) ROME IV IBS criteria of recurrent abdominal pain at least 1 day per week in the past 3 months that is associated with 2 or more of defecation, onset associated with a change in frequency of stool, or change in form of the stool; (3) have a diagnosis of IBS by a health care provider; (4) report moderate to severe anxiety or depression (Generalized Anxiety Disorder-7 [GAD-7] score of >10 [27]; Patient Health Questionnaire-9 [PHQ-9] score of >10 [28]); and (5) be able to read and write in English. Participants were excluded if they had a first-degree relative with colorectal cancer before the age of 60 years or had multiple “red flag” symptoms (ie, loss of 10 or more pounds without trying, blood in stool, or anemia). Patients completed a web-based screening questionnaire to assess their eligibility. All patients who expressed thoughts of hurting themselves were immediately directed to contact the National Suicide Prevention Lifeline.

Health care providers were recruited from primary care and gastroenterology clinics in Washington State. Individuals were eligible to participate if they self-reported caring for more than 3 patients with IBS per month. Health care providers from primary care clinics were recruited through the WWAMI (Washington, Wyoming, Alaska, Montana, and Idaho) region Practice and Research Network, a practice-based research network of primary care clinics and clinical organizations. Health care providers from Seattle gastroenterology clinics were recruited through purposeful convenience sampling. Health care providers received emails regarding the study and self-identified if they met the criteria of caring for at least 3 individuals with IBS per month.

### Description of the CSM Intervention at the Start of the Study

The CSM intervention was developed as a comprehensive approach to improving quality of life and reducing abdominal pain among individuals with IBS [15-17]. The intervention is delivered in 8 individual sessions, with sessions lasting 60 minutes. Participants had up to 12 weeks to complete the 8 sessions, to allow for unexpected events. Participants can elect to complete the intervention in-person, over the telephone, or through a mixture of telephone and in-person sessions since there is no difference in intervention effectiveness between in-person and telephone modalities [16]. Each participant receives a paper-bound “IBS Managing Symptoms Workbook,” which includes information, worksheets, and homework assignments. Additionally, participants received CD audio recordings of the relaxation exercises. The CSM intervention includes content such as healthy thought patterns, problem-solving, healthy eating, and relaxation. Additionally, the intervention addresses practical topics such as sleep, travel, eating out, and physical intimacy. Participants receive verbal and written instructions regarding the use of the manual.

### Measures

An overview of the measures by participant group (patients and health care providers) is presented in Table S1 in Multimedia Appendix 1.

#### Demographics

Age, sex, and race were assessed for patients and providers. For patients, the IBS subtype of constipation, diarrhea, or mixed was reported. Anxiety was measured using the GAD-7 questionnaire, which asks how often participants have been bothered by a list of 7 symptoms over the past 2 weeks [27]. Depression was measured with the PHQ-9, which asks participants how often they are bothered by 9 problems [28]. For both anxiety and depression measures, response options are on a 4-point scale, including “not at all,” “several days,” “more than half the days,” and “nearly every day.” Health care providers answered questions on the type of provider, years of working experience, and number of patients with IBS cared for per month.

#### Anticipated Acceptability, Appropriateness, and Feasibility

Anticipated acceptability, appropriateness, and feasibility were each measured with 4 items [26]. Participants responded on a 5-point Likert scale from 1 (completely disagree) to 5 completely agree. A higher score indicates greater agreement. Acceptability measures anticipate responsiveness to adopting a new implementation plan. Appropriateness measures the anticipated suitability of the intervention and the perceived fit of the intervention. Feasibility measures the anticipated likelihood of implementing the intervention.

#### Anticipated Usability

The anticipated usability of the CSM intervention was assessed using the Intervention Usability Scale, which has 10 items and was derived from the System Usability Scale [9,29]. Participants respond from “strongly disagree” to “strongly agree.” The scale ranges from 0 to 100. A score above 68 is considered average; a score below 68 is considered below-average usability.

### Procedures

Institutional review board approval was obtained from the University of Washington (IRB# 00009463) before participant recruitment. All individuals were provided with a description of the risks and benefits of participating in the research study as well as an explanation that they could stop participating at any time. Individual interviews were conducted with patients and health care providers. For this study, participants provided feedback on the intervention content and format overall without participating in individual intervention sessions. The second phase of this study (data not reported in this manuscript) focused on obtaining feedback on the intervention redesign of the first 3 intervention sessions.

Patients and health care providers were asked questions using a semistructured interview guide. For example, introductory questions were asked about symptoms (patients) and the type of practice (health care providers). Next, both patients and health care providers were shown an outline of the current CSM content and completed a card sorting activity to categorize the intervention content into 3 categories (“most helpful,” “seems okay,” or “least helpful”). Additionally, the current CSM intervention format was described (eg, in-person and telephone-delivered intervention that included a paper-based workbook); we asked for patients’ and health care providers’ thoughts on the intervention content outline and format and how it could best be designed to integrate into their lives and promote adherence to the intervention. At the end of the interview, patients and health care providers were sent postinterview participatory design session questionnaires regarding the anticipated acceptability, appropriateness, feasibility, and usability of the intervention.

### Data Analysis and Integration

Integration in quantitative and qualitative methods occurred through merging [25]. Quantitative and qualitative data were initially analyzed separately and brought together for analysis. For qualitative data, the interview recordings were transcribed. The 2 authors (KK and PLY) coded 2 transcripts from health care providers and 2 transcripts from patients to develop the coding scheme and reach a consensus using a framework approach [30]. The coding scheme was guided by the research questions to understand anticipated barriers and facilitators to acceptability, appropriateness, feasibility, and usability. Each coder then proceeded to code half of the interviews. Any discrepancies were discussed, and consensus was reached. The codes and results were presented to the entire team for further discussion. The mixed methods findings are presented within the text and highlight how the quantitative and qualitative findings align and show a confirmation of the findings, as well as those that are disparate and demonstrate discordance in findings [25]. By integrating the qualitative and quantitative data, we expanded our insights on which intervention components were acceptable and feasible and which needed modification. Mixed methods enabled us to gain new insights into the data, particularly by assessing numeric data to further explore themes from the qualitative interviews where usability or feasibility was lowest.

### Ethical Considerations

This study was reviewed by the University of Washington Institutional Review Board (IRB# 00009463). All participants provided verbal consent before the interview. Data are presented as deidentified and do not include links to participant characteristics to protect the privacy and confidentiality of the research participants. Participants received a US $50 gift card for participating in the study.

## Results

### Demographics

A total of 12 patients completed the qualitative interview (Table 1). Patients had a mean age of 36.8 (SD 12.2) years and were predominantly female. Overall, 42% (n=5) were currently receiving psychological therapy for anxiety or depression. A total of 14 health care providers completed the interview; half (7/14) were primary care providers, and half (7/14) specialized in gastroenterology. Professional roles included 7 physicians, 1 physician’s assistant, and 6 nurse practitioners. One provider did not complete the questionnaires. Provider experience ranged from 4 to 26 years. On average, providers cared for 26 (SD 20; range 3-80) patients with IBS per month. Interviews lasted between 27 and 43 minutes for health care providers (mean 35, SD 5 minutes) and between 32 minutes and 1 hour and 11 minutes for patients (mean 47, SD 12 minutes).

**Table 1:** Characteristics of patients with irritable bowel syndrome and comorbid anxiety or depression and health care providers.

		Patients (n=12)	Health care providers (n=14)
Age (years), mean (SD)		36.8 (12.2)	38.8 (5.8)
**Sex, n (%)**			
	Male	3 (25)	3 (23)
	Female	9 (75)	10 (77)
**Race, n (%)**			
	African American	1 (8)	0 (0)
	Asian	2 (16)	5 (38)
	White	9 (75)	7 (54)
	Other	1 (8)	1 (8)

### Anticipated Acceptability, Appropriateness, and Feasibility

#### Acceptability

Mean acceptability was 4.0 (SD 0.8) out of 5 by patients and 4.4 (SD 0.5) out of 5 by providers, indicating that on average, the current CSM intervention content and format were acceptable (Table S2 in Multimedia Appendix 1 for individual acceptability items).

The qualitative results confirm the intervention was acceptable through its comprehensive nature, in which patients could try different components and see what works for them (Table 2 for qualitative quotes). Participants reported that, given the gut-brain interaction that exists, a comprehensive approach to management of both gastrointestinal and anxiety or depressive symptoms was an important component. Patients scored acceptability lower than health care providers, particularly for the item that asks if the intervention is appealing. Patients identified that several of the intervention topics were familiar, especially those who have struggled to manage their IBS for many years. Health care providers focused on individual intervention components such as access and literacy, cost or insurance coverage, and culture or race that could be potential barriers to patients engaging in a self-management intervention like the CSM. The card sorting activity identified content that was of higher priority to participants. Patients found content related to sleep, traveling, and trigger foods most helpful, whereas providers found content related to relaxation, introduction to IBS, sleep, and trigger foods most helpful.

**Table 2:** Qualitative findings among 12 patients with irritable bowel syndrome and comorbid anxiety or depression and 14 primary care and gastroenterology health care providers.

Theme	Illustrative qualitative quotes
Acceptability	“I like the fact that there’s a wide variety of things; I feel confident that at least 1 or 2 of these things from the 7 kind of content weeks would be helpful.” [Patient 7] “I do think something like this could fit into my day-to-day life, because... it would give me some type of structure.” [Patient 8] “I really think this looks like a very comprehensive plan to address holistically what may be contributing to people who have irritable bowel syndrome.” [Provider 3] “I think improving accessibility, such that it’s one that I can give to [all] sort[s] of patients, regardless of their insurance status, regardless of where they live, or sort of their profession, would be good.” [Provider 8]
Appropriateness	“I feel like it would keep me more accountable, and it would give me somebody I can talk through everything with instead of just trying to figure it out on my own.” [Patient 11] “I think just having that intentionality and having structure is really important if someone wants to make a change.” [Patient 4] “So, I’ve done that in my therapy with a psychologist, but my therapist doesn’t know very much about IBS, and my doctor that knows about IBS, I just see them like for 15 minutes every 3 months or something. So, it would be nice to have someone who is aware of the integration of those things.” [Patient 7] “When you’re doing something yourself and it doesn’t rely on a medication and this gives people a little bit of power. It gives them structure.” [Provider 12] “I think that it also gives some accountability in terms of, ‘Did you do these exercises?’ ‘Did you bring your record?’ and these kinds of things.” [Provider 8] “When you’re depressed or anxious and when your body feels like it’s turning against you, which is what a lot of people with IBS I hear being said to me, it gives you a facet of control. When you’re doing something yourself and it doesn’t rely on a medication and this gives people a little bit of power. It gives them structure.” [Provider 10]
Feasibility	“I guess the part that might be difficult is just making sure someone actually does it, and sticking with it, which is the hard part.” [Patient 4] “I just think that somebody new to [IBS] would be more apt to get into this versus somebody who’s been through all this; they’d be like, ‘I’ve done all this stuff already.’” [Patient 5] “I’m curious, but also, I’m skeptical. I don’t know why. Just because I feel like I’ve tried so many things and I’m like, ‘Really? Fiber is going to be the thing?’ Maybe I just have more to learn.” [Patient 10] “I think the hard part is having a person that’s motivated enough to actually go through and do this on their own... unless there’s some accountability.” [Provider 12] “I think if they go in for counseling, they have more time to do CBT-type stuff. They have more time to talk to the patient about it. Whereas in primary care we don’t always have that kind of time, but I think if it’s something small and short that I could give them during the visit and then they can work on it.” [Provider 2]
Usability	“I’d like it with an app, something that’s visual on the app as well as verbal. I’d like some types of video content to actually show me certain tasks for working through planning out certain things, as well as verbalized communication.” [Patient 8] “In an e-course-esque environment I think would be really helpful or an app, if that’s a possibility.” [Patient 10] “If you construct your own. Build your own, I don’t know, Amazon cart, I don’t like the bundle.” [Patient 1] “I’d rather do it by myself and if there was somebody after the fact that wanted to check up on me for 5 minutes and say, how did it go? Do you have any questions? Did you have any concerns? Did it work?” [Patient 2] “I have a very ger[iatric] heavy panel, which certainly would not do well with an app and need kind of person kind of contacting them on a weekly basis in some shape or form phone call or something. Whereas I could definitely see my more hyper-focused, got a lot of stuff going on, needing it more as an app with an alert that pops up on their phone that says, ‘Hey, it’s time to work on your skill for today. Let’s set aside 15 minutes to do this,’ or whatever.” [Provider 1] “Just a very brief: Patient’s doing well. Patient does not seem to be progressing. Patient is not participating. They haven’t returned any of their journals.” [Provider 7] “Afterwards as a summary, was this overall sort of useful or which parts of it did you find use in? And, so then I know what are your residual symptoms that we can sort of work on and address because I think it’s also hard to see the clear benefit right away can sometimes take a while even with patients. Once it’s even kicked in, they have... for them to start suddenly realizing so many months down the road, ‘Actually my symptoms are doing a lot better. This used to be something I would think about all the time, and now it’s kind of rare.’” [Provider 8]

#### Appropriateness

Mean appropriateness was rated by patients as 4.0 (SD 0.7) and by providers as 4.2 (SD 0.5) out of 5. The qualitative data confirmed the quantitative finding by indicating that the intervention was appropriate because it provided accountability, support, and structure. This was especially important, as many patients felt they had tried multiple other strategies on their own through a trial-and-error approach and viewed the addition of a support person as very helpful and important. Patients discussed accountability within the context of having someone help them navigate and talk through their experiences. Health care providers also identified the importance of accountability, although they discussed accountability within the context of patients completing assignments and activities.

Patients and health care providers also addressed the appropriateness of the intervention in relation to patients with IBS who had comorbid anxiety or depression. Health care providers noted that structure was especially important as individuals with IBS and comorbid anxiety or depression often feel that a lot of things are out of their control. Even individuals who already received psychological therapy for anxiety or depression (n=5) saw the benefit of integrating IBS and mental health. The integration of an intervention that could address symptoms of IBS along with anxiety and depression was viewed positively by patients.

#### Feasibility

Mean feasibility was rated by patients as 3.9 (SD 0.7) and by providers as 3.9 (SD 0.7) out of 5. Among patients, the qualitative findings confirmed the quantitative findings. Patients identified specific situations that could influence feasibility, such as a lack of motivation to participate or “stick with” the intervention. Another factor affecting motivation is that many individuals with IBS and comorbid anxiety or depression have already tried multiple strategies to manage their symptoms. Several of the participants felt that components of the intervention were familiar and were perhaps better suited for newly diagnosed individuals. Due to having tried multiple previous strategies, some participants were skeptical that an intervention could truly help manage their symptoms. Yet, they were still interested in trying it due to experiencing symptoms. Patients primarily focused on the intervention feasibility.

Among health care providers, the quantitative and qualitative findings were discordant; the quantitative feasibility score was positive; however, the qualitative portion identified multiple barriers to implementation within a clinic setting. Similar to patients, health care providers also identified patient lack of motivation as a barrier to the intervention. Health care providers felt their clinic visits were so short that they did not have time to review the CSM self-management approaches. Health care providers’ comments regarding feasibility were focused on the feasibility of implementing the intervention in a health care clinic setting (see Perceived Usability section).

### Perceived Usability

#### Overview

The mean score for the Intervention Usability Scale was 52.5 (SD 14.5) for patients and 45.6 (SD 11.6) for health care providers, which fell below the average usability cutoff of 68. Usability conversations provided confirmation that the intervention needed to be revised and focused on improving the delivery of the intervention, the time demands of the intervention, and integration into health care settings. Textbox 1 presents a summary of recommended changes.

**Summary of recommended changes.**

- Patients were interested in moving through the intervention content at their own pace, but they still prefer a professional to check-in with for questions.
- Patients preferred the intervention content in a digital format.
- Reduce the face-to-face time required by providers to increase the likelihood that the intervention is adopted in clinical settings.
- Make the tracking (food, sleep, and symptoms) required by the intervention easier to do.
- Create content summarizing patient progress through the intervention to facilitate communication with their provider when they meet.

#### Delivery of Intervention

The original CSM was designed for in-person or telephone delivery with a paper workbook. Both patients and health care providers discussed the importance of continuing to have a person, either a health care provider or a peer, involved in the intervention. Patients, in particular, discussed the delivery of intervention content in online formats such as apps and e-courses where they could review content independently with weekly check-ins. All but 1 patient desired weekly check-ins. Some patients preferred web-based check-ins to be with an expert in IBS, whereas others noted that it may be helpful to have a peer mentor because they do not know many people with IBS. Regardless of who delivers the intervention (ie, health care provider or peer), the most important characteristic was someone who was skilled and knowledgeable in IBS. Health care providers said the delivery method depended on the age of the population. Some health care providers discussed the potential benefits of using videos to present information and demonstrate skills. However, most discussions with health care providers focused on in-person intervention delivery.

#### Intervention Time Demands

Patients had a variety of opinions regarding the length of the 8-week CSM intervention and the daily time commitment for practicing skills. Patients who preferred a shorter intervention typically identified content that was not applicable or of interest to them. For some, 8 weeks seemed reasonable, whereas others identified that 8 weeks may not be enough time to obtain results. Health care providers identified that the CSM, as originally designed, required more time to deliver the content than the providers were able to fit into their current practices. This creates an implementation barrier to incorporating the therapy into clinics.

#### Integration Into Health Care Settings

Health care providers thought the easiest integration into health care settings was for the providers to introduce the intervention to the patient and have another health care professional (eg, nurse or social worker) who was an expert in IBS deliver the intervention. Health care providers desired feedback on the patients’ progression through the intervention, such as a summary of symptoms, strategies that worked, and an overview of patient engagement. Most providers preferred a brief end-of-intervention update. Patients also preferred to learn about the intervention from their primary care provider or gastroenterologist.

## Discussion

### Overview

Overall, individuals with IBS and comorbid anxiety or depression, as well as health care providers, found the content and format of the CSM intervention acceptable and appropriate; however, challenges were identified related to anticipated feasibility and usability. The qualitative findings expanded the quantitative findings for acceptability and appropriateness. For feasibility, patient qualitative findings expanded the quantitative findings, whereas for providers, the qualitative findings indicated barriers to feasibility while the quantitative findings indicated feasibility. Overall, participants reported the intervention was comprehensive and provided structure, accountability, and support. However, participants warned that engagement in the intervention would be influenced by time, motivation, literacy, culture, and cost, in addition to a variety of usability issues (improving the delivery of the intervention, time demands of the intervention, and integration into health care settings). Addressing the anticipated acceptability, feasibility, appropriateness, and usability of the CSM intervention has the potential to influence key barriers to implementation and uptake among those with IBS and comorbid anxiety or depression.

### Acceptability

Previous research has noted that patients with medically unexplained symptoms and comorbid anxiety or depression may have less favorable cognitive behavioral therapy outcomes [31]. Thus, our approach of human-centered design methods to elicit feedback from patients with IBS and comorbid anxiety or depression may serve as 1 method to create interventions to address the unique needs of this population. Specifically, patients with IBS and comorbid anxiety or depression discussed the importance of structure and support in completing the intervention. They also mentioned the importance of integrating IBS and mental health as previous health care encounters had focused independently on either IBS or mental health but did not take a holistic approach to symptoms. Recent evidence has highlighted the benefits of integrated care approaches, which include a team comprising gastroenterologists, nurses, dietitians, psychiatrists, hypnotherapists, and behavioral therapists. Individuals with IBS who were randomized to an integrative care arm had greater reductions in global symptoms as well as IBS symptom severity compared to those in the standard care group [32].

### Appropriateness

Patients and health care providers both identified the importance of support from a person throughout the intervention. It is unclear if the desire for a support person is unique to patients with comorbid IBS and mental health. For instance, a meta-analysis found that computer-assisted cognitive behavioral therapy for depression in primary care is effective if clinicians offer modest support (60-194 minutes) throughout the intervention (7-16 weeks) [33]. In this study, patients preferred to review the content and practice independently and have someone available to follow up with them. Additionally, health care providers indicated that in-person sessions would be preferred but acknowledged that, with the COVID-19 pandemic, telemedicine visits could also be useful. Although a review article highlighted the effectiveness of primary care provider–delivered self-management interventions, this study highlights the time limitations of clinicians in delivering such an intervention [34]. Even if primary care providers do not deliver the intervention, there is a need for integrated care approaches so that primary care providers can receive feedback on their patients progress through the intervention. Future research should examine innovative methods to integrate comprehensive interventions into primary care, gastroenterology, and other health care settings.

### Feasibility

Patients indicated that the intervention may be best suited for newly diagnosed individuals to promote self-management earlier in the disease course. Thus, additional research is needed to understand if the intervention effects differ based on time since diagnosis or time since symptom onset. Health care providers had varied opinions regarding the feasibility of the intervention, specifically highlighting barriers to implementation. Yet few studies have focused on implementation strategies within the population of patients with IBS and anxiety or depression overlap. Implementation frameworks such as the Consolidated Framework for Implementation Research [35], the Practical, Robust Implementation, and Sustainability Model [36], and the Reach Effectiveness Adoption Implementation and Maintenance framework [37] can be used to guide such research.

### Usability

Both patients and health care providers identified several ways to improve the usability of the intervention. Patients emphasized the benefits of accessing intervention content on the internet and being able to track and monitor symptoms. Health care providers identified age as a factor influencing intervention delivery methods, although this theme did not arise among the small sample of patients aged between 20 and 59 years. Previous research has indicated that older adults may avoid using technology due to fear of confirming negative stereotypes [38]. Thus, considerations should be made for understanding the specific technology needs of older adults with IBS and comorbid anxiety or depression and designing accessible systems that benefit all patients.

Patients had a variety of opinions regarding the length of the intervention. Patient differences such as disease severity, previous intervention experiences, or lifestyle may influence intervention length preferences. A previous study by Lackner et al [6] identified no statistically significant differences in the proportion of patients with IBS responding to a 10-week standard cognitive behavioral therapy (87.5%) compared to a brief 4-week cognitive behavioral therapy (80%, *P*>.55) [6]. Thus, there is a need to identify ways to tailor the intervention length based on the content participants are familiar with and their tolerance for intervention length.

### Limitations

Strengths of the study include incorporating the perspectives of both patients and health care providers into the intervention evaluation. Using implementation science and a human-centered design approach provided an established framework to elicit feedback regarding the intervention. Yet, this study has several limitations. Patients and health care providers were recruited during the COVID-19 pandemic. Patients were recruited on the internet and therefore may have different characteristics than those typically obtained in primary care and gastroenterology settings, such as greater levels of computer literacy and comfort with technology-delivered interventions. Additionally, we did not have access to clinical records for patients. It is possible that selection bias may have occurred such that health care providers with a large number of patients with IBS were more likely to participate in the study and had different barriers than health care providers seeing fewer patients with IBS. Another limitation is that patients and providers did not complete the intervention but rather provided feedback on the overall content and intervention; therefore, additional implementation barriers may be found when using the intervention.

### Conclusions

Patients and health care providers reported the CSM intervention was acceptable and appropriate but identified several potential feasibility and usability challenges. Thus, before applying the intervention among a population of individuals with IBS and comorbid anxiety or depression, there is a need to modify intervention delivery methods, consider the length of the intervention, and address the best methods of implementing in the clinic setting. Considerations should be made to improve the ease of tracking, allow participants to move through the intervention at their own pace, and provide a summary of patient progress through the intervention. Future work will assess whether addressing feasibility and usability leads to the anticipated improvements in implementation and adoption.

## Appendix

Multimedia Appendix 1 Study Measures Completed by Patients with Irritable Bowel Syndrome and Comorbid Anxiety and/or Depression and Healthcare Providers.

Multimedia Appendix 2 Individual items from the acceptability, appropriateness, and feasibility measures.

## Abbreviations

CSM: comprehensive self-management
EBPI: evidence-based practice intervention
GAD-7: General Anxiety Disorder-7
IBS: irritable bowel syndrome
PHQ-9: Patient Health Questionnaire-9

## Data Availability

The data sets generated and analyzed during this study are available from the corresponding author on reasonable request.
