# Supplementary material for: Formative Evaluation of a Comprehensive Self-Management Intervention for Irritable Bowel Syndrome, Comorbid Anxiety, and Depression: Mixed Methods Study
Source: JMIR Form Res. 2024 Jan 31;8:e43286. doi: 10.2196/43286 (PMC10867748; doi:10.2196/43286)
Supplement: Multimedia Appendix 1 [file formative_v8i1e43286_app1.docx]

|  | **Patients** | **Healthcare Providers** |  |
| --- | --- | --- | --- |
| **Demographics** | | | |
| Age | X | X |  |
| Gender | X | X |  |
| Race | X | X |  |
| IBS Subtype (constipation, diarrhea, mixed) | X |  |  |
| Anxiety (GAD-7) | X |  |  |
| Depression (PHQ-9) | X |  |  |
| Type of provider |  | X |  |
| Years working experience |  | X |  |
| Number of IBS patients cared for per month |  | X |  |
| **Anticipated Acceptability, Appropriateness, and Feasibility** | | | |
| Acceptability of Intervention Measure | X | X |  |
| Intervention Appropriateness Measure | X | X |  |
| Feasibility of Implementation Measure | X | X |  |
| **Anticipated Usability** | | | |
| Intervention Usability Scale | X | X |  |
| One-on-one interview | X | X |  |

**Table S1:** Study Measures Completed by Patients with Irritable Bowel Syndrome and Comorbid Anxiety and/or Depression and Healthcare Providers

Patients were individuals who had irritable bowel syndrome and comorbid anxiety and/or depression
