# Supplementary material for: Formative Evaluation of a Comprehensive Self-Management Intervention for Irritable Bowel Syndrome, Comorbid Anxiety, and Depression: Mixed Methods Study
Source: JMIR Form Res. 2024 Jan 31;8:e43286. doi: 10.2196/43286 (PMC10867748; doi:10.2196/43286)
Supplement: Multimedia Appendix 2 [file formative_v8i1e43286_app2.docx]

**Table S2:** Individual items from the acceptability, appropriateness, and feasibility measures

|  | **Patients**  **(n=12)** | **Health Care Providers**  **(n=14)** |
| --- | --- | --- |
|  | **M (SD)** | **M (SD)** |
| **Acceptability of Intervention Measure** |  |  |
| This CSM Intervention meets my approval. | 4.0 (1.0) | 3.9 (0.8) |
| This CSM Intervention is appealing to me. | 3.7 (1.0) | 4.4 (0.7) |
| I like this CSM Intervention. | 4.0 (0.9) | 4.4 (0.9) |
| I welcome this CSM Intervention. | 4.2 (0.7) | 4.4 (0.8) |
| **Intervention Appropriateness Measure** |  |  |
| This CSM Intervention seems fitting | 4.0 (0.7) | 4.2 (0.8) |
| This CSM Intervention seems suitable. | 4.1 (0.8) | 4.2 (0.8) |
| This CSM Intervention seems applicable. | 4.0 (0.8) | 4.2 (0.8) |
| This CSM Intervention seems like a good match. | 4.0 (0.7) | 3.9 (0.9) |
| **Feasibility of Implementation Measure** |  |  |
| This CSM Intervention seems implementable. | 4.0 (0.9) | 3.8 (0.8) |
| This CSM Intervention seems possible. | 4.0 (0.9) | 4.2 (0.6) |
| This CSM Intervention seems doable. | 4.0 (0.7) | 4.0 (0.7) |
| This CSM Intervention seems easy to use. | 3.8 (0.9) | 3.5 (1.1) |
